# Multi-Objective Optimization Based Multi-Bernoulli Sensor Selection for Multi-Target Tracking

**DOI:** 10.3390/s19040980

**Published:** 2019-02-25

**Authors:** Yun Zhu, Jun Wang, Shuang Liang

**Affiliations:** 1 National Laboratory of Radar Signal Processing, Xidian University, Xi’an 710071, China; wangjun@xidian.edu.cn; 2 Key laboratory of Intelligent Perception and Image Understanding of Ministry of Education, Xi’an 710071, China; s.leung615@outlook.com

**Keywords:** multi-target tracking, sensor management, random finite set, multi-objective optimization

## Abstract

This paper presents a novel multi-objective optimization based sensor selection method for multi-target tracking in sensor networks. The multi-target states are modelled as multi-Bernoulli random finite sets and the multi-Bernoulli filter is used to propagate the multi-target posterior density. The proposed method is designed to select the sensor that provides the most reliable cardinality estimate, since more accurate cardinality estimate indicates more accurate target states. In the multi-Bernoulli filter, the updated multi-target density is a multi-Bernoulli random finite set formed by a union of legacy tracks and measurement-updated tracks. The legacy track and the measurement-updated track have different theoretical and physical meanings, and hence these two kinds of tracks are considered separately in the sensor management problem. Specifically, two objectives are considered: (1) maximizing the mean cardinality of the measurement-updated tracks, (2) minimizing the cardinality variance of the legacy tracks. Considering the conflicting objectives simultaneously is a multi-objective optimization problem. Tradeoff solutions between two conflicting objectives will be derived. Theoretical analysis and examples show that the proposed approach is effective and direct. The performance of the proposed method is demonstrated using two scenarios with different levels of observability of targets in the passive sensor network.

## 1. Introduction

Tracking of multi-target in sensor networks is an important but challenging task for radar, sonar and other surveillance systems [1]. Most traditional multi-target tracking formulations involve explicit associations between measurements and targets, based on the assumption that target states and models are known a priori. When it is difficult to know the true underlying model, joint Bayesian parameter estimation and model selection have been extensively studied within the Monte Carlo (MC) methodology [2,3]. However, due to communication and real-time constraints of the sensor network, the system may need to select a subset of sensors to report high quality target-related measurements [4,5]. Sensor selection (also named as sensor management) is inherently an optimal nonlinear control problem. Different from the classical control problem, it is embedded in highly complex multi-target systems. In multi-target systems, the number of targets and their individual states are random and unknown. Besides, the measurements are subject to missed detections and false alarms. Multi-target tracking under these circumstances is nontrivial and the difficulty is further compounded in the sensor selection problem [6].

In general, sensor selection is developed based on Bayesian multi-target filtering. Mahler’s Finite set statistics (FISST) provides an elegant Bayesian filtering formulation based on random finite set (RFS) theory and has attracted a large amount of attention in recent years [7]. The probability hypothesis density (PHD) [8] and cardinalized PHD (CPHD) [9] filters are two popular approaches within FISST. They propagate moment approximations of the RFS of states in time and avoid the combinatorial problem arising from data association. Another type of FISST is the multi-target multi-Bernoulli (MeMBer) filter whose recursion propagates the approximate posterior multi-target density. However, the MeMBer filter was shown to overestimate the cardinality (number of targets). To solve this problem, a modified filter named Cardinality-Balanced MeMBer (CBMeMBer) was proposed in [10]. For these FISST filters, tractable solution methodologies can be provided by Sequential Monte Carlo (SMC) and Gaussian mixture implementations.

Several sensor-management solutions have been recently formulated within the FISST multi-target filtering. In general, an objective function is developed as the sensor-management criterion and is used to quantify the effect of each admissible command. A common approach is to measure the information gained via updating the predicted density and a typical objective function is the Rényi divergence between the predicted and updated density. In [11], the Rényi reward function was associated with the multi-target Bayes filter. However, this approach is computationally intractable even for a small number of targets. To increase the computation efficiency, the Rényi divergence was investigated within the PHD filtering scheme under the assumption of independent and identically distributed cluster [12]. Then, the work in [13] demonstrated that using the same Rényi reward function, the CBMeMBer filter based strategy performs better than the PHD filter based methods. However, the Rényi divergence function has no analytic solution and its computation needs MC sampling of the updated multi-target multi-Bernoulli distribution, resulting in great computational burden. An alternative information divergence measure is the Cauchy-Schwarz (CS) divergence [14]. An analytic expression for the CS divergence was developed as the reward function in [15,16]. However, the divergence is derived in some abstract mathematical sense but its practical interpretation is unclear. Due to the limitations of the divergence function mentioned above, it is desirable to manage the sensors by optimizing a task-driven objective function. In [17,18], the cost function was defined in terms of the cardinality variance, which can be computed analytically and enables an efficient sensor management. In [19], the authors proposed to minimize the dispersion of the multi-target posterior, quantified based on a multi-target estimation error metric. In presence of numerous sensors, [20] introduced a guided search to solve the multi-dimensional discrete optimization problem embedded in multi-sensor control.

This paper formulates the sensor management problem as a multi-objective optimization problem with two different objective functions, to improve the tracking performance while maintaining low computation cost. The CBMeMBer recursion is used as the multi-target filter because its SMC implementation accommodates non-linear non-Gaussian models and allows reliable and inexpensive extraction of state estimates. In contrast, the PHD and CPHD recursions require clustering to extract state estimates from the particle population, which is expensive and unreliable. Another advantage of the SMC implementation of the CBMeMBer recursion is that it allows the state-dependent probability of detection, which makes the filter applicable to more general detection profiles. At each time step, the multi-Bernoulli parameter set for the updated multi-target density is formed by the union of the multi-Bernoulli parameter sets for the legacy tracks and measurement-updated tracks. Nevertheless, the legacy track and the measurement-updated track are updated in different manners and have different physical meaning. To the best of the authors’ knowledge, there is no literature considering the legacy tracks and measurement-updated tracks separately in solving the sensor management problem. In this paper, the objective functions are developed as the mean cardinality of the measurement-updated tracks and the cardinality variance of the legacy tracks, both of which can be computed analytically. Maximizing the mean cardinality of the measurement-updated tracks while minimizing the cardinality variance of the legacy tracks is conflicting and is modelled as a multi-objective optimization problem. The multi-objective problem gives a set of sensor selection solutions that reveal different trade-offs between conflicting objectives. The preferred solution–the one most desirable to the decision maker or the designer–is selected from the Pareto optimal set [21]. In the following, the proposed method is named as the mean-variance optimization (MVO) method. Simulation results for two challenging scenarios validate the advantages of the MVO method over the competitive methods.

The paper is organized as follows. In Section 2, the necessary background on multi-target Bayesian filtering is presented and the CBMeMBer recursion is briefly reviewed. Then, motivations and definitions of the proposed objective functions are presented and details of the multi-objective optimization procedure are given in Section 3, followed by numerical studies in Section 4. Finally, Section 5 concludes the paper.

## 2. Background

### 2.1. Multitarget Bayesian Framework

An RFS is intuitively a random (spatial) point pattern, in which the number of points is random and the points themselves are random and unordered. In multi-target systems, not only does the number of targets vary randomly in time, but also the measurements are subject to missed detections and false alarms. This means that the set of target states and of measurements are both inherently RFSs. Suppose that at time *k*, there are $n_{k}$ target states $x_{k}^{\left(1\right)},x_{k}^{\left(2\right)},\ldots ,x_{k}^{\left(n_{k}\right)}$ belonging to the state space $X\subseteq R^{n_{x}}$ and $m_{k}$ measurements $z_{k}^{\left(1\right)},z_{k}^{\left(2\right)},\ldots ,z_{k}^{\left(m_{k}\right)}$ taking values in the observation space $Z\subseteq R^{n_{z}}$. The finite sets of targets and observations are treated as the multi-target state and multi-target observation, respectively

(1) $$X_{k}=\{x_{k}^{\left(1\right)},x_{k}^{\left(2\right)},\ldots ,x_{k}^{\left(n_{k}\right)}\}\in F(X)$$

(2) $$Z_{k}=\{z_{k}^{\left(1\right)},z_{k}^{\left(2\right)},\ldots ,z_{k}^{\left(m_{k}\right)}\}\in F(Z)$$

where F(X) and F(Z) denote the space of finite subsets of X and Z, respectively. 

The multi-target filtering problem can be formulated in the Bayesian filtering framework. Let $\pi_{k}(X_{k}|Z_{1:k})$ denote the multi-target posterior density at time *k*. The multi-target Bayes recursion propagates $\pi_{k}(X_{k}|Z_{1:k})$ according to two main steps: time prediction and measurement update

(3) $$\pi_{k|k-1}(X_{k}|Z_{1:k-1})=\int f_{k|k-1}(X_{k}|X)\pi_{k-1}(X|Z_{1:k-1})\delta X$$

(4) $$\pi_{k}(X_{k}|Z_{1:k})=\frac{g_{k}(Z_{k}|X_{k})\pi_{k|k-1}(X_{k}|Z_{1:k-1})}{\int g_{k}(Z_{k}|X)\pi_{k|k-1}(X|Z_{1:k-1})\delta X}$$

where $f_{k|k-1}(\cdot |\cdot )$ is the multi-target transition density that encapsulates the underlying models of target motions, births and deaths; $g_{k}(\cdot |\cdot )$ is the multi-target likelihood that encapsulates the underlying models of detections and false alarms. Note that the integrals in (3) and (4) are not ordinary integrals, but are FISST set integrals, which have no analytic solution and computationally intractable in general. 

### 2.2. Cardinality Balanced MeMBer filter

The CBMeMBer recursion is an approximation to the full multi-target Bayes recursion (3) and (4) using multi-Bernoulli RFSs [10]. A Bernoulli RFS X on X has probability 1−r of being empty, and probability *r* of being a singleton distributed according to a probability density p (defined on X). The cardinality distribution of the Bernoulli RFS X is a Bernoulli distribution with parameter *r*, while its probability density is [7]

(5) $$\pi (X)=\left\{ \begin{array}{ll} 1-r & X=\emptyset \\ rp(x) & X=\{x\} \end{array}\right.$$

A multi-Bernoulli RFS is defined as the union of *M* independent Bernoulli RFSs $X=\cup_{i=1}^{M}X^{\left(i\right)}$. The probability density is

(6) $$\pi (\emptyset )=\prod_{j=1}^{M}\left(1-r^{\left(j\right)}\right)$$

and

(7) $$\pi (\{x_{1},\ldots ,x_{n}\})=\pi (\emptyset )\sum_{1\leq i_{1}\neq \ldots \neq i_{n}\leq M}\prod_{j=1}^{n}\frac{r^{\left(i_{j}\right)}p^{\left(i_{j}\right)}(x_{j})}{1-r^{\left(i_{j}\right)}}$$

Thus, the multi-Bernoulli RFS can be completely denoted as $\pi ={\left\{(r_{}^{\left(i\right)},p_{}^{\left(i\right)})\right\}}_{i=1}^{M}$.

If at time *k*−1, the posterior density is of the form $\pi_{k-1}={\left\{(r_{k-1}^{\left(i\right)},p_{k-1}^{\left(i\right)})\right\}}_{i=1}^{M_{k-1}}$. Then, the predicted density at time *k* is also a multi-Bernoulli and is given by

(8) $$\pi_{k|k-1}={\left\{(r_{p,k|k-1}^{\left(i\right)},p_{p,k|k-1}^{\left(i\right)})\right\}}_{i=1}^{M_{k-1}}\cup {\left\{(r_{\Gamma ,k}^{\left(i\right)},p_{\Gamma ,k}^{\left(i\right)})\right\}}_{i=1}^{M_{\Gamma ,k}}$$

where ${\left\{(r_{\Gamma ,k}^{\left(i\right)},p_{\Gamma ,k}^{\left(i\right)})\right\}}_{i=1}^{M_{\Gamma ,k}}$ are parameters of births at time *k* and ${\left\{(r_{p,k|k-1}^{\left(i\right)},p_{p,k|k-1}^{\left(i\right)})\right\}}_{i=1}^{M_{k-1}}$ are used to express the survival ones which are computed as

(9) $$r_{p,k|k-1}^{\left(i\right)}=r_{k-1}^{\left(i\right)}\langle p_{k-1}^{\left(i\right)},p_{S,k}\rangle$$

(10) $$p_{p,k|k-1}^{\left(i\right)}(x)=\frac{\langle f_{k|k-1}(x|\cdot ),p_{k-1}^{\left(i\right)},p_{S,k}\rangle }{\langle p_{k-1}^{\left(i\right)},p_{S,k}\rangle }$$

where the inner product $\langle p_{k-1}^{\left(i\right)},p_{S,k}\rangle =\int p_{k-1}^{\left(i\right)}(x)p_{S,k}(x)dx$, $p_{S,k}(x)$ is the survival probability of x and $f_{k|k-1}(\cdot |\cdot )$ is the single target transition density.

Let us denote the predicted density by the form $\pi_{k|k-1}={\left\{(r_{k|k-1}^{\left(i\right)},p_{k|k-1}^{\left(i\right)})\right\}}_{i=1}^{M_{k|k-1}}$, then the posterior density can be approximated as follows:

(11) $$\pi_{k}\approx {\left\{(r_{L,k}^{\left(i\right)},p_{L,k}^{\left(i\right)})\right\}}_{i=1}^{M_{k|k-1}}\cup \{(r_{U,k}(z),p_{U,k}(\cdot ;z))\}z\in Z_{k}$$

where ${\left\{(r_{L,k}^{\left(i\right)},p_{L,k}^{\left(i\right)})\right\}}_{i=1}^{M_{k|k-1}}$ corresponds to the legacy tracks and $\left\{(r_{U,k}(z),p_{U,k}(\cdot ;z))\right\}$ are the measurement-updated tracks. The existence probability and the spatial distribution are given by

(12) $$r_{L,k}^{\left(i\right)}=r_{k|k-1}^{\left(i\right)}\frac{1-\langle p_{k|k-1}^{\left(i\right)},p_{D,k}\rangle }{1-r_{k|k-1}^{\left(i\right)}\langle p_{k|k-1}^{\left(i\right)},p_{D,k}\rangle }$$

(13) $$p_{L,k}^{\left(i\right)}(x)=p_{k|k-1}^{\left(i\right)}(x)\frac{1-p_{D,k}(x)}{1-\langle p_{k|k-1}^{\left(i\right)},p_{D,k}\rangle }.$$

(14) $$r_{U,k}(z)=\frac{\sum_{i=1}^{M_{k|k-1}}\frac{r_{k|k-1}^{\left(i\right)}\left(1-r_{k|k-1}^{\left(i\right)}\right)\langle p_{k|k-1}^{\left(i\right)},\psi_{k,z}\rangle }{{\left(1-r_{k|k-1}^{\left(i\right)}\langle p_{k|k-1}^{\left(i\right)},p_{D,z}\rangle \right)}^{2}}}{\kappa_{k}(z)+\sum_{i=1}^{M_{k|k-1}}\frac{r_{k|k-1}^{\left(i\right)}\langle p_{k|k-1}^{\left(i\right)},\psi_{k,z}\rangle }{1-r_{k|k-1}^{\left(i\right)}\langle p_{k|k-1}^{\left(i\right)},p_{D,z}\rangle }}$$

(15) $$p_{U,k}(x;z)=\frac{\sum_{i=1}^{M_{k|k-1}}\frac{r_{k|k-1}^{\left(i\right)}}{1-r_{k|k-1}^{\left(i\right)}}p_{k|k-1}^{\left(i\right)}(x)\psi_{k,z}(x)}{\sum_{i=1}^{M_{k|k-1}}\frac{r_{k|k-1}^{\left(i\right)}}{1-r_{k|k-1}^{\left(i\right)}}\langle p_{k|k-1}^{\left(i\right)},\psi_{k,z}\rangle }$$

(16) $$\psi_{k,z}(x)=g_{k}(z|x)p_{D,k}(x)$$

where $g_{k}(\cdot |x)$ is the single target measurement likelihood function, $p_{D,k}(x)$ is the state dependent probability of detection and $\kappa_{k}(\cdot )$ is the intensity of the clutter. 

The SMC implementation of the CBMeMBer recursion is adopted in this paper. At time *k*, the probability density $p_{k}^{\left(i\right)}$ is approximated by a set of particles

(17) $$p_{k-1}^{\left(i\right)}(x)=\sum_{j=1}^{L_{k-1}^{\left(i\right)}}w_{}^{\left(i,j\right)}\delta_{x^{\left(i,j\right)}}(x)$$

where $\delta_{a}(x)$ is the Dirac delta function concentrated at a, $L_{k-1}^{\left(i\right)}$ is the number of particles for *i*th Bernoulli component, and $w_{}^{\left(i,j\right)}$ is the normalized weight of *j*th particle from *i*th Bernoulli component. To reduce the number of particles, at each time step hypothesized tracks with existence probabilities below a threshold *P* are discarded. For the remaining hypothesized tracks, a maximum of $L_{\max}$ and minimum of $L_{\min}$ particles are imposed on each hypothesized track. For more details of the SMC implementation, the reader is referred to [10].

## 3. Multi-Bernoulli Sensor Selection via Multi-Objective Optimization

As mentioned earlier, this paper focuses on solving the sensor selection problem in the context of choosing the best selection command among a finite number of admissible commands. We formulate this problem in the partially observed Markov decision process (POMDP) framework [22]. In a POMDP, the multi-target dynamics is modeled as a Markov process, but there is no direct access to current states and information can only be obtained from the past measurements [23]. The POMDP used in this paper is represented as

(18) $$\Psi =\left\{X_{k},S,f_{k|k-1}\left(X_{k}|X_{k-1}\right),g_{k}\left(Z_{k}|X_{k}\right),\vartheta \left(X_{k-1},s,X_{k}\right)\right\}$$

where S denotes a finite set of sensors for selection and $\vartheta \left(X_{k-1},s,X_{k}\right)$ is an objective function that measures a reward or cost for transition from $X_{k-1}$ to $X_{k}$ given that the sensor s∈S is selected. The purpose of sensor selection is to find the optimal sensor $\overset{*}{s}$ which optimizes the statistical mean of the objective function $\vartheta \left(X_{k-1},s,X_{k}\right)$ over all possible measurements [24]:

(19) $$\overset{*}{s_{k}}=\underset{s\in S}{\arg\min}/\underset{s\in S}{\mathrm{argmax}}\left\{E_{Z_{k}\left(s\right)}\left[\vartheta \left(X_{k-1},s,X_{k}\right)\right]\right\}$$

Note that the general formulation of a POMDP involves a p-step future decision process, whereas, in this paper we only consider a single step ahead (myopic) policy. 

### 3.1. Objective Functions Proposal 

Recall that the updated multi-target density is formed by two different sets of the multi-Bernoulli parameters that correspond to the legacy tracks [the first term in (11)] and the measurement-updated tracks [the second term in (11)]. Moreover, the legacy tracks and measurement-updated tracks are updated in different manners and have different physical meaning. Thus, the sensor selection in this paper is posed as a multi-objective optimization problem which consists of two objective functions corresponding to the legacy tracks and measurement-updated tracks, respectively. Assume that the updated multi-target density for sensor *s* at time *k* is a multi-Bernoulli RFS denoted as

(20) $${\left\{(r_{k}^{\left(i\right)}(s),p_{k}^{\left(i\right)}(s))\right\}}_{i=1}^{M_{k}\left(s\right)}={\left\{(r_{L,k}^{\left(i\right)}(s),p_{L,k}^{\left(i\right)}(s))\right\}}_{i=1}^{M_{k|k-1}}\cup \{(r_{U,k}(z(s)),p_{U,k}(\cdot ;z(s)))\},z(s)\in Z_{k}(s)$$

where $Z_{k}(s)$ is the set of measurements collected by sensor *s* at time *k*. Motivations and definitions of the two objective functions are as follows. 

The first objective function is the mean cardinality of the measurement-updated tracks. Maximizing of this function has the effect of maximizing the number of detected targets and also has the side-effect of improving the localization accuracy of such target as time goes on. At time *k*, the mean cardinality of the measurement-updated tracks for sensor *s* is defined as

(21) $$N_{U,k|k}\left(s\right)=\sum_{z(s)\in Z_{k}(s)}r_{U,k}(z(s))$$

Some methods have been proposed for maximizing the number of detected targets in sensor management using the PHD and CPHD filters. In [25,26] the posterior expected number of targets was employed as a reward function, which is obtained by integrating the intensity density of the PHD filter. Later, [27] shows that this reward function can be extended to the CPHD filter. Since the CPHD filter is more accurate than the PHD filter, the CPHD-based approach to sensor management produces more accurate results than the PHD-based approach. We use the CBMeMBer filter because it outweighs the PHD and CPHD filters in the SMC implementation. 

On the other hand, the second objective function, the cardinality variance of the legacy tracks, aims to improve the accuracy of the number of legacy tracks. The cardinality variance of the legacy tracks for sensor *s* at time *k* is given by

(22) $$\sigma_{L,k|k}^{2}\left(s\right)=\sum_{i=1}^{M_{k|k-1}}r_{L,k}^{\left(i\right)}(s)(1-r_{L,k}^{\left(i\right)}(s))$$

The cardinality variance is a measure of the uncertainty embedded in the estimated tracks. Therefore, we propose to use the statistical cardinality variance of the legacy tracks as the objective function, which tends to minimize the error in the number of the legacy tracks. Both the mean cardinality and the cardinality variance can be computed analytically and cheaply. Therefore, these two objective functions can provide an efficient solution for sensor management.

The objective functions are developed with parameters of the updated multi-Bernoulli distribution. However, the updated distribution depends on the measurement set, which is a function of the chosen sensor-management command. Theoretically, all possible measurement sets should be used to update the distribution, which is computationally expensive. To reduce the computational complexity, the predicted ideal measurement set (PIMS) approach [25] is used. First, the estimated number and states of targets are obtained from the predicted multi-target density. Then, a measurement is generated for each target under ideal conditions of no clutter, no measurement noise and perfect detection. The expectation term in (19) does not appear in the proposed objective functions, as the PIMS approach is used instead of sampling or averaging in measurement space. 

### 3.2. Illustrative Examples

To distinguish the proposed objective functions from the mean cardinality of the posterior density and its variance, we also give the definitions and notations for the latter. At time *k*, the mean cardinality of the posterior density (given by (20)) and its variance for sensor *s* are defined as

(23) $$N_{k|k}(s)=\sum_{i=1}^{M(s)}r_{k}^{\left(i\right)}(s)$$

(24) $$\sigma_{k|k}^{2}(s)=\sum_{i=1}^{M(s)}r_{k}^{\left(i\right)}(s)(1-r_{k}^{\left(i\right)}(s))$$

where $N_{k|k}(s)$ and $\sigma_{k|k}^{2}(s)$ denote the mean cardinality and the cardinality variance of the posterior density, respectively. In the following, two indicative examples are used to illustrate why the measurement-updated tracks and legacy tracks should be considered separately, but not using the posterior density as a whole. In the first example, the mean cardinality of the posterior density, $N_{k|k}(s)$, is used as the objective function. In the second one, the cardinality variance of the posterior density, $\sigma_{k|k}^{2}$, is regarded as the objective function.

**Example 1:** For sensors $s_{1}$ and $s_{2}$, the existence probabilities of the measurement-updated tracks are (0.7,0.7) and (0.5,0.6), respectively. Besides, the existence probabilities of the legacy tracks are 0.1 and 0.5, respectively. For $s_{1}$ and $s_{2}$, the mean cardinality of the posterior density is $N_{k|k}\left(s_{1}\right)=0.7+0.7+0.1=1.5$ and $N_{k|k}\left(s_{2}\right)=0.5+0.6+0.5=1.6$, respectively. Using the mean cardinality of the posterior density as the objective function, we should select $s_{2}$, since $s_{2}$ returns a larger value of the objective function. 

For $s_{1}$ and $s_{2}$, the mean cardinality of the measurement-updated tracks are $N_{U,k|k}\left(s_{1}\right)=0.7+0.7=1.4$ and $N_{U,k|k}\left(s_{2}\right)=0.5+0.6=1.1$, respectively. Therefore, $s_{1}$ can detect more targets than $s_{2}$. Furthermore, the variance of the legacy tracks for $s_{1}$ and $s_{2}$ are $\sigma_{L,k|k}^{2}\left(s_{1}\right)=0.1\times (1-0.1)=0.09$ and $\sigma_{L,k|k}^{2}\left(s_{2}\right)=0.5\times (1-0.5)=0.25$, respectively. Thus, $s_{1}$ can provide more reliable number of the legacy tracks than $s_{2}$. Based on the above analysis, it would be better to select $s_{1}$. Using the mean cardinality of the posterior density as the objective function may not lead to meaningful choice of actions. 

**Example 2:** For sensors $s_{1}$ and $s_{2}$, the existence probabilities of the measurement-updated tracks are (0.1,0.4) and (0.6,0.9), respectively. Besides, the existence probabilities of the legacy tracks are (0.3,0.6) and (0.7,0.1), respectively. For $s_{1}$ and $s_{2}$, the variance of the posterior density is $\sigma_{k|k}^{2}(s_{1})=0.1\times (1-0.1)+0.4\times (1-0.4)+0.3\times (1-0.3)+0.3\times (1-0.3)=0.75$ and $\sigma_{k|k}^{2}\left(s_{2}\right)=0.6\times (1-0.6)+0.9\times (1-0.9)+0.7\times (1-0.7)+0.6\times (1-0.6)=0.78$, respectively. Using the variance of the posterior density as the objective function, we should select $s_{1}$, since it returns a smaller value of the objective function. 

For $s_{1}$ and $s_{2}$, the mean cardinality of the measurement-updated tracks are $N_{U,k|k}\left(s_{1}\right)=0.1+0.4=0.5$ and $N_{U,k|k}\left(s_{2}\right)=0.6+0.9=1.5$, respectively. Therefore, $s_{2}$ can detect more targets than $s_{1}$. Furthermore, the variance of the legacy tracks for $s_{1}$ and $s_{2}$ are $\sigma_{L,k|k}^{2}\left(s_{1}\right)=0.3\times (1-0.3)+0.6\times (1-0.6)=0.45$ and $\sigma_{L,k|k}^{2}\left(s_{2}\right)=0.7\times (1-0.7)+0.1\times (1-0.1)=0.3$, respectively. Thus, $s_{2}$ can provide more reliable number of the legacy tracks than $s_{1}$. Based on the above analysis, it would be better to select $s_{2}$. 

### 3.3. Multi-Objective Optimization

This paper intends to account for both the mean cardinality of the measurement-updated tracks and the cardinality variance of the legacy tracks. Maximizing the mean cardinality $N_{U,k|k}(s)$ while minimizing the cardinality variance $\sigma_{L,k|k}^{2}(s)$ is a multi-objective problem. To formulate the problem as a general optimization, we minus the mean cardinality $-N_{U,k|k}\left(s\right)$, translating the maximization into the minimization. At time *k*, the objectives for sensor s∈S are as follows

(25) $$\mathrm{Minimize}\;f_{1}\left(s\right)=-N_{U,k|k}\left(s\right)=-\sum_{z(s)\in Z_{k}(s)}r_{U,k}(z(s))$$

(26) $$\mathrm{Minimize}\;f_{2}\left(s\right)=\sigma_{L,k|k}^{2}\left(s\right)=\sum_{i=1}^{M_{k|k-1}}r_{L,k}^{\left(i\right)}(s)(1-r_{L,k}^{\left(i\right)}(s))$$

The mathematical description of the multi-objective optimization problem is given as

(27) $$\begin{array}{l} \mathrm{Minimize}\;F\left(s\right)={\left[f_{1}^{}\left(s\right),f_{2}^{}\left(s\right)\right]}^{T}\\ \mathrm{Subject}\;\mathrm{to}\;s\in S \end{array}$$

where F(s) is the objective vector and s is a decision variable within a parameter space S. 

The utopian solution is the solution that is optimal for all objectives. However, it does not generally exist since $f_{1}\left(s\right)$ and $f_{2}\left(s\right)$ can be conflicting. Classical approach usually converts the multi-objective problem into a single optimization problem by aggregating all objectives in a weighted function. This approach has several limitations, such as the requirement of a priori knowledge about the relative importance of the objectives and the solution may not be attainable unless the search space is convex [21]. 

In fact, there is a set of solutions for the multi-objective optimization problem, which represent different trade-offs between the objectives. A solution $s_{1}$ is said to Pareto-dominate the solution $s_{2}$ in a minimization context, if and only if [21]

(28) $$\forall i\in \{1,2\},f_{i}\left(s_{1}\right)\leq f_{i}\left(s_{2}\right),\mathrm{and}\;\exists j\in \{1,2\},f_{j}\left(s_{1}\right)<f_{j}\left(s_{2}\right)$$

A solution is Pareto optimal if and only if there does not exist another solution that dominates it. The set of all Pareto optimal solutions is called the Pareto optimal set. The corresponding objective vectors form the Pareto front. 

It is necessary to select one particular solution from the Pareto-front which can yield the desired trade-off between the conflicting objectives. We take a simple strategy and use the Euclidean distance to find the compromise solution [28]. The utopia point $F^{*}$ of the multi-objective problem is defined as

(29) $$F^{*}={\left[f_{1}^{*},f_{2}^{*}\right]}^{T}$$

where $f_{1}^{*}$ and $f_{2}^{*}$ are the minima of objectives $f_{1}^{}\left(s\right)$ and $f_{2}^{}\left(s\right)$, respectively.

(30) $$f_{j}^{*}=\underset{s}{\min}\left\{f_{j}^{}\left(s\right)|s\in S\right\},\;j\in \left\{1,2\right\}$$

The point in the Pareto optimal set which is closest to the utopia point is regarded as the compromise solution. The shortest Euclidean distance of a point $F\left(s\right)={\left[f_{1}^{}\left(s\right),f_{2}^{}\left(s\right)\right]}^{T}$ from the utopia point can be determined as follows

(31) $$d_{Euc}=\underset{s\in S}{\min}\sqrt{{\left(f_{1}^{*}-f_{1}^{}\left(s\right)\right)}^{2}+{\left(f_{2}^{*}-f_{2}^{}\left(s\right)\right)}^{2}}$$

**Figure 1:**
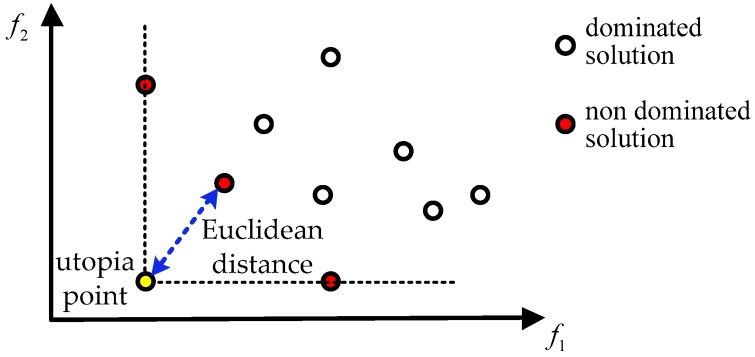
Illustration of the multi-objective problem. The red dots form the Pareto front, the yellow dot denotes the utopia point, and the blue dashed line stands for the shortest Euclidean distance.

In this paper, the number of sensors to be selected is assumed known a priori and hence the generation of the Pareto front is straightforward. Figure 1 gives an illustration of the multi-objective problem, where the number of all possible solutions is 10 and 3 solutions are Pareto optimal.

## 4. Simulations

**Figure 2:**
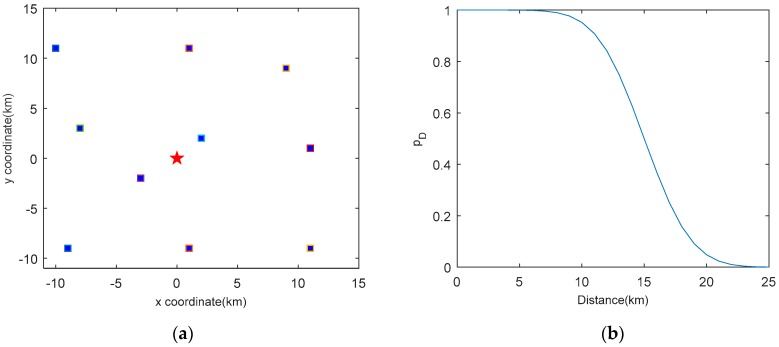
System setup: (**a**) The locations of receivers (squares) and the transmitter (star); (**b**) Probability of detection as a function of the distance from a receiver.

In this section, we demonstrate the performance of the proposed MVO method with two scenarios and compare it with the Rényi divergence based method of [13] and the cardinality variance strategy reported in [17]. Both scenarios are studied in a passive sensor network whose structure is borrowed from [29]. In the network, one transmitter and ten receivers are placed in the *x*–*y* plane as shown in Figure 2a. During the target tracking process, the receivers are selected automatically at each time step. All receivers j=1,2,…,10 are characterized by identical measurement noise and the sampling interval is fixed to $T_{s}=10\;s$. The probability of detection is modeled as follows [29]

(32) $$p_{D}^{\left(j\right)}(x_{k})=1-\phi (\left\|p_{k}-r^{j}\right\|;\alpha ,\beta )$$

where $p_{k}={\left[x_{k},y_{k}\right]}^{T}$ is the target position, $r^{j}$ is the position of receiver j, $d_{k,j}=\left\|p_{k}-r^{j}\right\|$ is the distance between the target and the receiver, and $\phi (d;\alpha ,\beta )=\int_{-\infty }^{d}N(v;\alpha ,\beta )dv$ is the Gaussian cumulative distribution function with α=15 km and $\beta ={(3\;\mathrm{km})}^{2}$. Figure 2b plots the probability of detection as a function of the distance between the receiver and the target. Note that with the increase of the distance from the receiver, the probability of detection of the passive sensor decreases quickly [30]. The poor performance of the probability of detection makes this a difficult problem of sensor management for target tracking, in which many state-of-the-art techniques would fail. 

**Figure 3:**
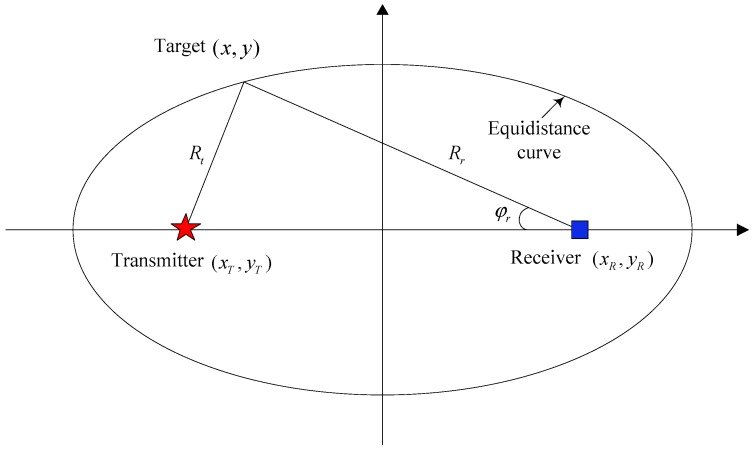
The geometry of a single transmitter-target-receiver pair. Locations of the transmitter and the receiver are shown with the red star and blue square, respectively. The equidistance curve can be plotted using the bistatic range $\rho =R_{t}+R_{r}$ and the target position is further determined using the bearing $\varphi_{r}$.

Two challenging scenarios are studied in the passive sensor network. In Scenario 1, a time varying number of targets turn through the scenario and the motion model is complex. The bistatic range (transmitter-target-receiver) and bearing measurements are used to guarantee full observability of the targets. In this case, the measurement is informative and hence only one receiver is selected at each time step. Scenario 2 is used to compare the performance of MVO method with other sensor management methods using the bistatic range only measurement. In this case, more than one receiver is selected at each time step to avoid the “ghost particles” caused by the high nonlinearity of the measurement model. For the selected receives, the posterior density is updated sequentially. For more details of the multi-Bernoulli filter with sequential update scheme, the reader is referred to [31]. The geometry of a single transmitter-target-receiver pair is illustrated in Figure 3, where the locations of the transmitter, the receiver and the target are $\left(x_{T},y_{T}\right)$, $\left(x_{R},y_{R}\right)$ and (x,y), respectively.

At each time step, $L_{\max}=1000$ and $L_{\min}=300$ particles per Bernoulli component is imposed. Pruning of Bernoulli component is performed with a threshold of P=10e−3. The survival probability is fixed to $p_{s}=0.99$. The birth process is a multi-Bernoulli RFS and all probabilities of existence are chosen at $r_{\Gamma }=0.02$. The tracking performance is evaluated using the optimal sub-pattern assignment (OSPA) error distance [32]. If $X=\{x_{1},x_{2},\cdots ,x_{n}\}$, $Y=\{y_{1},y_{2},\cdots ,y_{m}\}$, and n≥m, the OSPA distance is defined as

(33) $$d_{p,c}^{\mathrm{OSPA}}(X,Y):={\left(\frac{1}{n}\left(\underset{\pi \in \Pi_{n}}{\min}\sum_{i=1}^{m}d^{\left(c\right)}{\left(x_{i},y_{{}^{\pi (i)}}\right)}^{p}+c^{p}(n-m)\right)\right)}^{1/p}$$

where $d^{\left(c\right)}(x,y):=min(c,\left\|x-y\right\|)$, c>0 is the cut-off parameter, p≥1 is an order parameter, and π is a permutation function in the set of permutations $\Pi_{n}$. If n<m, $d_{p,c}^{\mathrm{OSPA}}(X,Y):=d_{p,c}^{\mathrm{OSPA}}(Y,X)$. For each scenario, the average error performances are obtained over 100 MC trials. All experiments are tested in Matlab R2010a and implemented on a computer with a 3.40 GHz processor.

### 4.1. Scenario 1

In this scenario, the non-linear bearing and bistatic range measurements are used to track a time varying number of targets. A nearly constant turn model is considered. The target state is denoted as $x_{k}={\left[{\tilde{x}}_{k}^{T},\omega_{k}\right]}^{T}$ where ${\tilde{x}}_{k}={\left[x_{k},{\dot{x}}_{k},y_{k},{\dot{y}}_{k}\right]}^{T}$ comprises the target position and velocity and $\omega_{k}$ is the turn rate. The state transition model is

(34) $${\tilde{x}}_{k}=F(\omega_{k-1}){\tilde{x}}_{k-1}+Gw_{k-1}$$

where

(35) $$F(\omega_{k-1})=\left[\begin{array}{cccc} 1 & \frac{\sin\omega T_{s}}{\omega } & 0 & -\frac{1-\cos\omega T_{s}}{\omega }\\ 0 & \cos\omega T_{s} & 0 & -\sin\omega T_{s}\\ 0 & \frac{1-\cos\omega T_{s}}{\omega } & 1 & \frac{\sin\omega T_{s}}{\omega }\\ 0 & \sin\omega T_{s} & 0 & \cos\omega T_{s} \end{array}\right]$$

(36) $$G=\left[\begin{array}{cc} \frac{T_{s}^{2}}{2} & 0\\ T_{s} & 0\\ 0 & \frac{T_{s}^{2}}{2}\\ 0 & T_{s} \end{array}\right]$$

**Figure 4:**
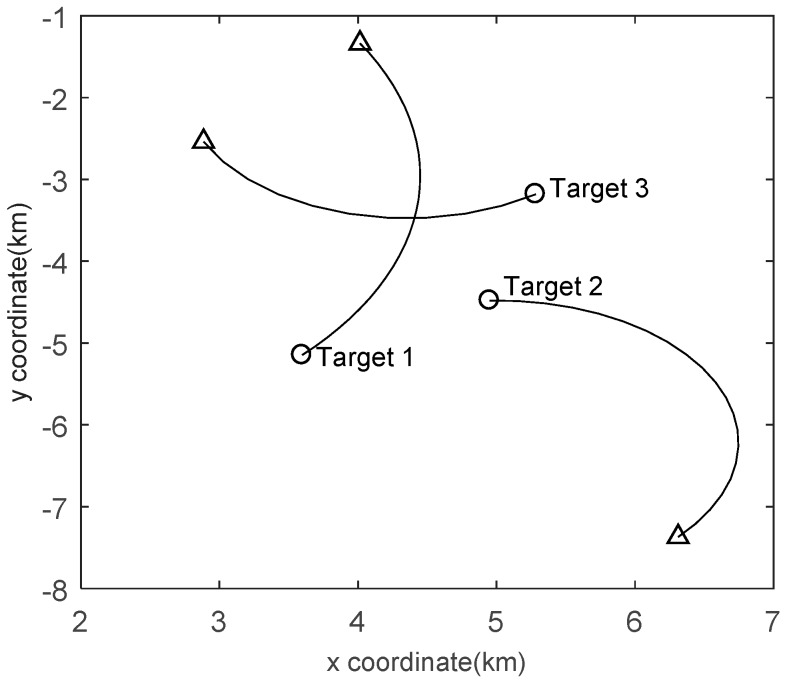
Target trajectories of Scenario 1 in the *x*/*y* plane. Start/Stop positions for each track are shown with ○/△.

$w_{k-1}\sim N(\cdot ;0,P)$ is a 2×1 independent and identically distributed Gaussian process noise vector with covariance $P=\sigma_{w}^{2}I_{2}$, where $\sigma_{w}=0.01\;m/s^{2}$ is the standard deviation of the target acceleration. A total of three targets appear in the surveillance area and the true trajectories of targets are shown in Figure 4, in which Target 1 is born at k=1, Target 2 is born at k=10 and Target 3 is born at k=20. If the target is detected by the receiver j located at $r^{j}={\left[x_{R}^{j},y_{R}^{j}\right]}^{T}$, then the measurement is a noisy bearing and bistatic range vector given by

(37) $$z_{k}^{j}=\left[\begin{array}{c} \varphi_{r}\\ \rho^{j} \end{array}\right]=\left[\begin{array}{c} \arctan\left(\frac{y_{k}-y_{R}^{j}}{x_{k}-x_{R}^{j}}\right)\\ \sqrt{\left(x_{k}-x_{{}_{T}})+(y_{k}-y_{{}_{T}}\right)}+\sqrt{\left(x_{k}-x_{R}^{j})+(y_{k}-y_{R}^{j}\right)} \end{array}\right]+\varepsilon_{k}^{j}$$

where $\varepsilon_{k}^{j}\sim N(\cdot ;0,R_{k})$, with $R_{k}=\mathrm{diag}([\sigma_{\varphi }^{2},\sigma_{\rho }^{2}])$, $\sigma_{\varphi }=(\pi /180)\;\mathrm{rad}$, and $\sigma_{\rho }=5\;m$. 

For the CBMeMBer filter, the birth process is a multi-Bernoulli RFS $\pi_{\Gamma }={\left\{\left(r_{\Gamma }^{},p_{\Gamma }^{\left(i\right)}\right)\right\}}_{i=1}^{3}$, where $p_{\Gamma }^{\left(i\right)}=N(x;m_{\Gamma }^{\left(i\right)},P_{\Gamma })$, $m_{\Gamma }^{\left(1\right)}={\left[3500,0,-5250,0,0\right]}^{T}$, $m_{\Gamma }^{\left(2\right)}={\left[4750,0,-4500,0,0\right]}^{T}$, $m_{\Gamma }^{\left(3\right)}={\left[5500,0,-3000,0,0\right]}^{T}$, and $P_{\Gamma }=\mathrm{diag}{\left({\left[50,50,50,50,6(\pi /180)\right]}^{T}\right)}^{2}$. The units are meters for *x* and *y* and meters per second for $\dot{x}$ and $\dot{y}$. The clutter measurements for each receiver are distributed uniformly over the region [−π,π] rad×[0,15000] m. The number of clutter measurements per scan is assumed to be Poisson distributed with the clutter intensity $\lambda_{c}=2\times 10^{-5}\;{\left(\mathrm{radm}\right)}^{-1}$ (giving an average of 2 clutter points per scan). To evaluate the performance of the MVO method, we compare it with the cardinality variance based method [17] and the Rényi detergency based method [13]. The Rényi detergency measures information gain between two densities and its parameter α tunes the emphasis on each of the two densities. In the simulations, the Rényi detergency based method is evaluated with α→1 and α=0.5, and the Rényi divergence becomes the Kullback–Leibler divergence and the Hellinger affinity, respectively [1]. To make the comparisons more meaningful, the PIMS strategy is used in all of the methods.

**Figure 5:**
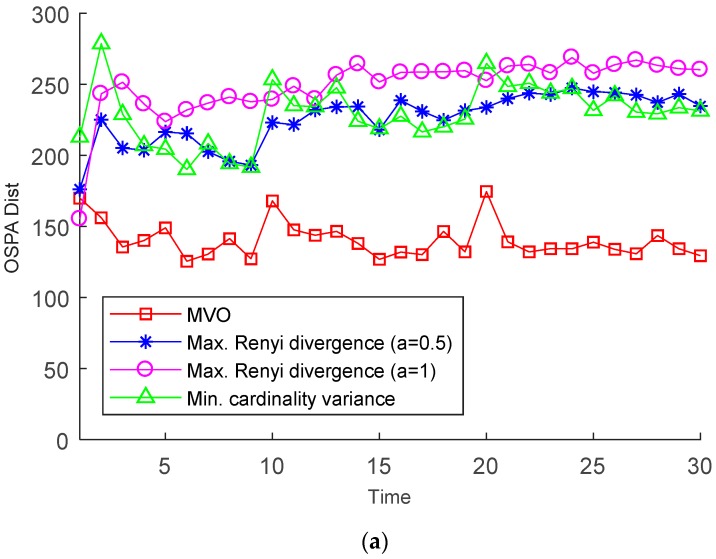

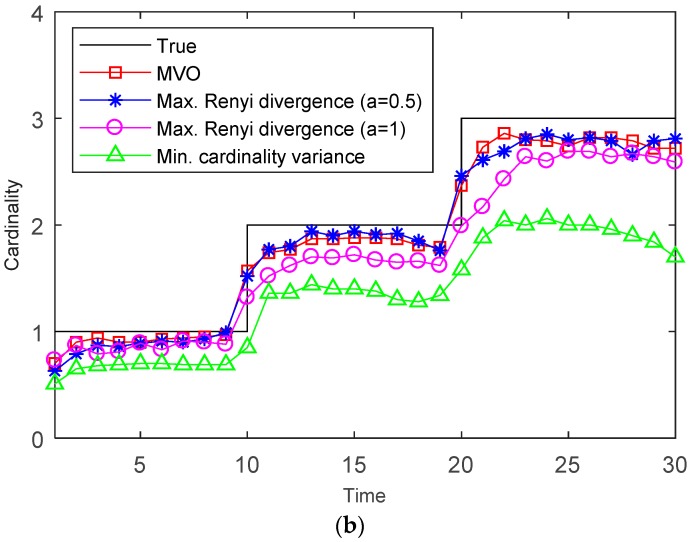
OSPA distances and cardinality estimates versus time: (**a**) OSPA distance; (**b**) Cardinality estimates.

The MC averages of the OSPA distance (for *p* = 1, c = 300 m) and the cardinality estimate are given in Figure 5. It can be observed that the MVO method performs better than the Rényi divergence based method. This is mainly because the cost function of the MVO method provides a better estimate of the target number and also tend to improve the localization accuracy of the detected targets. Since the measurements used to select the sensors are created in the ‘‘ideal’’ manner in PIMS, the Rényi detergency with α=0.5 provides the maximum discrimination between the two similar densities [1]. Thus, in our case, the Rényi detergency based method with α=0.5 performs than it with α→1. Using only the cardinality variance as the cost function, it is more easily to lose targets when the probability of detection is not satisfactory, as shown in Figure 5b. The cost function of the MVO method includes both the mean cardinality and the cardinality variance which can enhance the performance of sensor management. Thus, the MVO method outperforms the method reported in [17] in terms of the OSPA error. 

The average run time for a complete MC simulation are 77.73 s (Rényi divergence based method with α→1), 121.46 s (Rényi divergence based method with α=0.5), 67.33 s (cardinality variance based method), and 70.96 s (MVO method). The Rényi divergence based method is more time-consuming than other methods, because it has no analytic solution in general and requires sampling on the multi-target state space. The MVO method is slower than the cardinality variance based method. Since the mean cardinality is not considered in the cardinality variance based method, the miss-detections occur often when the probability of detection is not satisfactory, as seen in Figure 5b. Therefore, the cardinality variance based method works more efficiently but performs worse than the MVO method.

**Figure 6:**
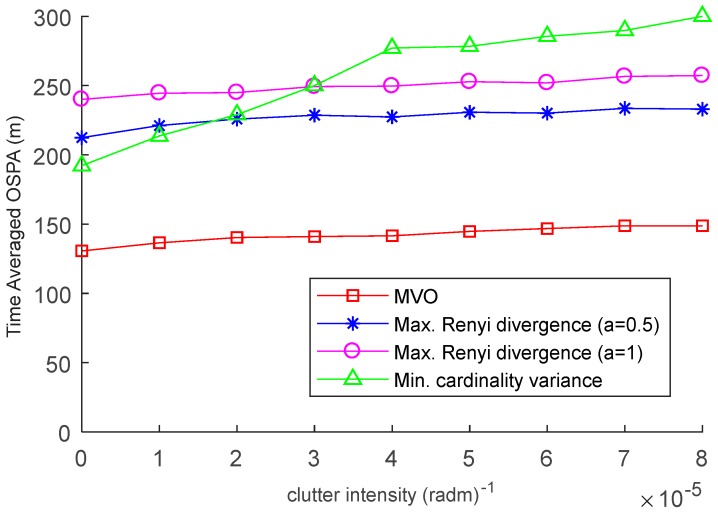
Time-averaged OSPA distances for varying clutter intensity.

In Figure 6, the time averaged OSPA distances over the duration of the scenario for the Rényi detergency based method, the cardinality variance based method and the MVO method are shown against clutter intensities from $\lambda_{c}=0\;{\left(\mathrm{radm}\right)}^{-1}$ to $\lambda_{c}=8\times 10^{-5}\;{\left(\mathrm{radm}\right)}^{-1}$. As expected, the miss-distances increase with higher clutter intensities. Moreover, with the increase of the clutter intensity, the performance of the cardinality variance based method degrades more quickly than other methods. This is primarily because the high clutter intensity and undesirable probability of detection together would lead to large cardinality errors of the cardinality variance based method. In contrast, the performance of the MVO method is stable and better than other methods under consideration.

### 4.2. Scenario 2

Instead of bearing and bistatic range measurements, we now consider bistatic range only measurements and assume no target birth or death. The target state variable is a vector of target position and velocity and is denoted as $x_{k}={\left[x_{k},y_{k},{\dot{x}}_{k},{\dot{y}}_{k}\right]}^{T}$. The single-target transition model is linear Gaussian specified by

(38) $$x_{k}=F_{k}x_{k-1}+u_{k}$$

with the transition matrix

(39) $$F=\left[\begin{array}{cccc} 1 & 0 & T_{s} & 0\\ 0 & 1 & 0 & T_{s}\\ 0 & 0 & 1 & 0\\ 0 & 0 & 0 & 1 \end{array}\right]$$

**Figure 7:**
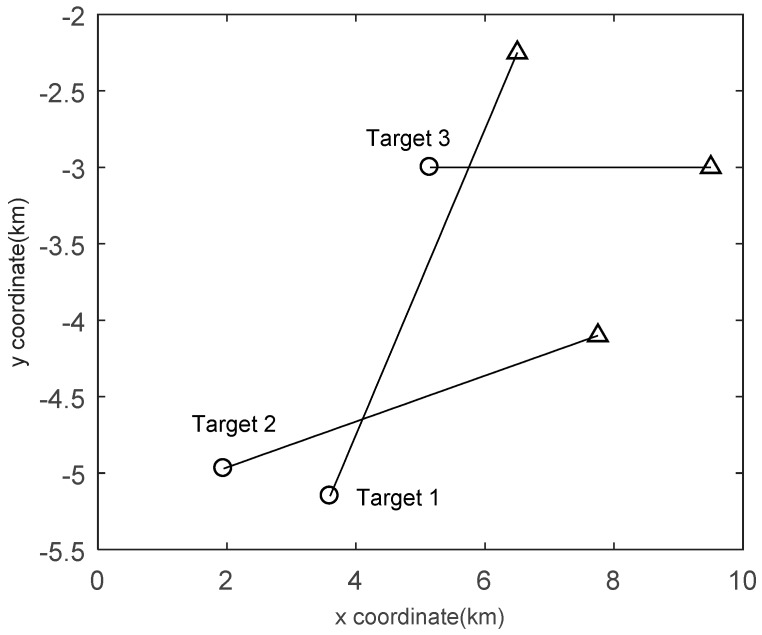
Target trajectories of Scenario 2 in the *x*/*y* plane. Start/Stop positions for each track are shown with ○/△.

The process noise ${\begin{array}{c} u \end{array}}_{k}$ is zero-mean Gaussian distributed with covariance

(40) $$Q=\sigma_{u}^{2}\left[\begin{array}{cccc} \frac{T_{s}^{3}}{3} & 0 & \frac{T_{s}^{2}}{2} & 0\\ 0 & \frac{T_{s}^{3}}{3} & 0 & \frac{T_{s}^{2}}{2}\\ \frac{T_{s}^{2}}{2} & 0 & T_{s} & 0\\ 0 & \frac{T_{s}^{2}}{2} & 0 & T_{s} \end{array}\right]$$

where $\sigma_{u}=0.01\;m/s^{2}$ is the standard deviation of the process noise. The true trajectories are shown in Figure 7.

The birth multi-Bernoulli RFS is denoted as $\pi_{\Gamma }={\left\{\left(r_{\Gamma }^{},p_{\Gamma }^{\left(i\right)}\right)\right\}}_{i=1}^{3}$, where $p_{\Gamma }^{\left(i\right)}=N(x;m_{\Gamma }^{\left(i\right)},P_{\Gamma })$, $m_{\Gamma }^{\left(1\right)}={\left[3500,10,-5250,10\right]}^{T}$, $m_{\Gamma }^{\left(2\right)}={\left[1750,20,-5000,3\right]}^{T}$, $m_{\Gamma }^{\left(3\right)}={\left[5000,15,-3000,0\right]}^{T}$, and $P_{\Gamma }=\mathrm{diag}{\left({\left[10,2,10,2\right]}^{T}\right)}^{2}$. The clutter measurements for each receiver are distributed uniformly over the region [0,15000] m with the clutter intensity $\lambda_{c}=1.33\times 10^{-4}\;{\left(m\right)}^{-1}$ (giving an average of 2 clutter points per scan). All other parameters are the same as those in the previous scenario. The bistatic range only measurements are much less informative, and result in a lower target ‘‘observability’. If only one receiver is selected at each time step and the same receiver is selected for some consecutive time steps, the remaining particles will not only from target but also include “ghost particles” and these “ghost particles” occupy a major part of overall particles until they are severely biased from real target state. As a result, the performances of all sensor management methods can be worse. To solve this problem, more than one receiver is selected at each time step and the posterior densities of the selected receivers are updated sequentially. For simplify, the number of receivers being selected at each time step is denoted by *S.*

**Figure 8:**
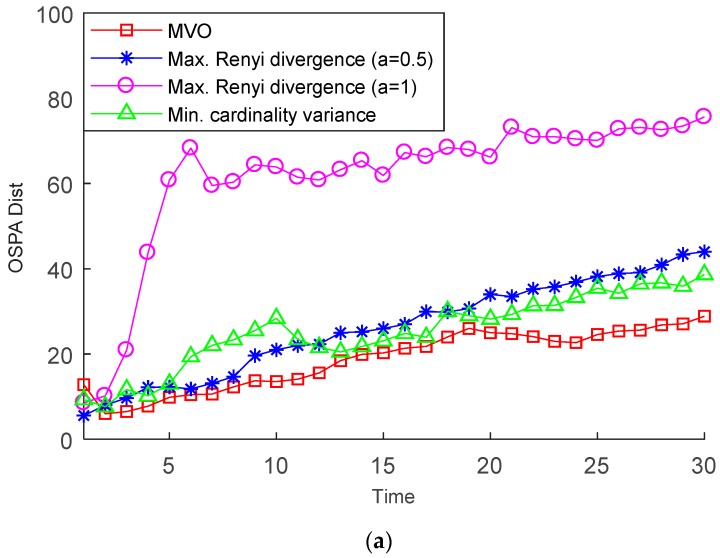

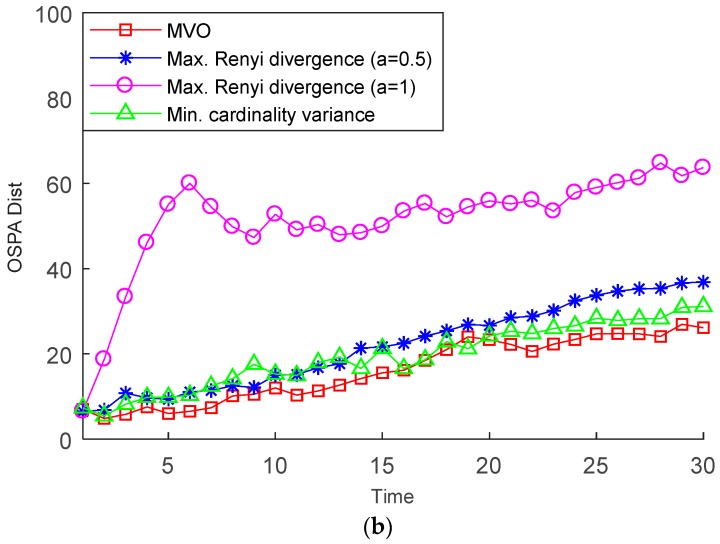
Mean OSPA error for different number of selected receivers: (**a**) S = 2; (**b**) S = 3.

We verify the performance of the MVO method using different *S*. Figure 8 shows the averaged OSPA distances (with parameters *p* = 1, *c* = 100 m) of the MVO method, the cardinality variance based method and the Rényi detergency based method for *S* = 2, 3. Due to the lack of observability of the full states, it is difficult to resolve the targets. To avoid this problem and track the multiple targets, the birth multi-Bernoulli RFSs are generated using the initial target states. Therefore, it can be seen that the OSPA distances of all methods are on a rising trend. Figure 8 demonstrates that, in accordance with our intuition, using more receivers at each time step *k* can effectively reduce the OSPA distances. Moreover, it appears that the MVO method outperforms other methods. Compared with Scenario 1, the cardinality variance based method performs better in Scenario 2. Thus, the “observability” of the targets and the target motions can be crucial factors in determining the performance of the sensor management method. 

## 5. Conclusions

A sensor-selection solution has been proposed to be employed within a multi-Bernoulli multi-target filter. At each step, the next sensor-selection command is chosen by solving a multi-objective problem. It’s easy to see that each objective function can be computed analytically, enabling a principled and efficient solution for the sensor selection problem. The performance of the proposed method was demonstrated in two challenging scenarios where a passive sensor network with low detection probability is used to track targets with different levels of observability. Simulation results demonstrated that the proposed sensor selection method performs better than the competing methods. Future work will apply the proposed multi-objective methodology to other more advanced filters such as the generalized multi-Bernoulli filter.
